# A Cellular Resolution Spatial Transcriptomic Landscape of the Medial Structures in Postnatal Mouse Brain

**DOI:** 10.3389/fcell.2022.878346

**Published:** 2022-05-17

**Authors:** Mengnan Cheng, Liang Wu, Lei Han, Xin Huang, Yiwei Lai, Jiangshan Xu, Shuai Wang, Mei Li, Huiwen Zheng, Weimin Feng, Zirui Huang, Yujia Jiang, Shijie Hao, Zhao Li, Xi Chen, Jian Peng, Pengcheng Guo, Xiao Zhang, Guangyao Lai, Qiuting Deng, Yue Yuan, Fangming Yang, Xiaoyu Wei, Sha Liao, Ao Chen, Giacomo Volpe, Miguel A. Esteban, Yong Hou, Chuanyu Liu, Longqi Liu

**Affiliations:** ^1^ College of Life Sciences, University of Chinese Academy of Sciences, Beijing, China; ^2^ BGI-Shenzhen, Shenzhen, China; ^3^ Laboratory of Integrative Biology, Guangzhou Institutes of Biomedicine and Health, Chinese Academy of Sciences, Guangzhou, China; ^4^ BGI College and Henan Institute of Medical and Pharmaceutical Sciences, Zhengzhou University, Zhengzhou, China; ^5^ State Key Laboratory for Zoonotic Diseases, Key Laboratory for Zoonosis Research of Ministry of Education, Institute of Zoonosis, College of Veterinary Medicine, Jilin University, Changchun, China; ^6^ Joint School of Life Sciences, Guangzhou Institutes of Biomedicine and Health, Guangzhou Medical University, Guangzhou, China; ^7^ Hematology and Cell Therapy Unit, IRCCS Istituto Tumori “Giovanni Paolo II”, Bari, Italy; ^8^ Bioland Laboratory (Guangzhou Regenerative Medicine and Health Guangdong Laboratory), Guangzhou, China

**Keywords:** spatial transcriptomics, stereo-seq, postnatal mouse brain development, single cell, brain region delineation and identification

## Introduction

Mammalian brain development is a complex process that starts from the neurulation of the early embryo to maturation and functionalization in postnatal brain development (Stiles and Jernigan, 2010). Major biological events that occur during brain development include cell proliferation, neurogenesis, cell migration, gliogenesis, and myelination, during which numerous cells are generated and migrate to specific regions to obtain corresponding functions (Jiang and Nardelli, 2016). These processes adhere to tight spatiotemporal regulation by extrinsic and intrinsic cues across different anatomical regions or layers (Martinez, 2001; Echevarria et al., 2003; Hodge and Hevner, 2011; Manuel et al., 2015). The disruption of gene expression or regulatory factors can lead to developmental dysfunction or neuropsychiatric diseases (Li et al., 2018; Parenti et al., 2020). Previous studies have mainly focused on the molecular features of specific regions; however, a complete understanding of brain development requires comprehensive characterization of cell types, molecular features, and spatial localization of each cell or gene at the whole-brain scale (Tiklova et al., 2019; Tan et al., 2021).

Mouse brain development can be broadly divided into two stages, embryonic and postnatal. During the embryonic stage, starting from embryonic day 10 (E10), a large number of neurons are generated by cell proliferation and neurogenesis. Within the first week after birth, numerous glial cells are generated, which are critical for neuronal synaptogenesis. By postnatal day 7 (P7), many neurons start forming synaptic connections to build the initial neural circuits of the brain (Han et al., 2009). Besides, some brain regions undergo dramatic changes during this period. For example, neurogenesis in the dentate gyrus of the hippocampus begins at E10 and reaches a maximum rate in the first two postnatal weeks (P0–P14) (Zhang et al., 2005). Granule cells of the cerebellum begin at E15 and undergo massive proliferation, peaking from P5 to P7 (Zhang et al., 2005; Carter et al., 2018; Kozareva et al., 2021). Therefore, it is significant to understand the molecular, cellular, and regional characteristics of this stage.

Single-cell and spatial transcriptomic technologies have greatly accelerated the understanding of cell type taxonomy, spatial localization, and functions in both adult and developing brains (Lein et al., 2017). For example, Zeisel et al. (2018) described the transcriptional features of 19 regions in the central nervous system and peripheral nervous system of adult mice, including the olfactory bulb, cortex, nucleus (striatum, hypothalamus, and so on), midbrain, and spinal cord. Some studies have focused on specific cortical regions of the adult brain in mice or humans (Lake et al., 2016; Tasic et al., 2016; Tasic et al., 2018). The current systematic molecular description of mouse brain development comes from the Allen Institute, which used the ISH approach (Thompson et al., 2014). Primate brain development has been systematically described which covers the stage from E40 to postnatal 48 months (Bakken et al., 2016). Whole-brain coverage of spatial transcriptome (via laser microdissection) and gene expression (via ISH) are also available for prenatal human brains (Miller et al., 2014; Ding et al., 2022). Furthermore, studies have focused on resolving the developmental features of the prenatal mouse brain, ranging from E7 to E18.5 (Di Bella et al., 2021; La Manno et al., 2021); however, a comprehensive study on the spatial organization of cell types at the maturation stage in the postnatal mouse brain is yet to be performed.

The rapid evolution of cutting-edge spatial transcriptomic technologies has provided new methods to decipher complex brains (Liao et al., 2021; Ortiz et al., 2021). Although FISH-based spatial technology has allowed the study of gene expression patterns in specific regions, the limited number of detected genes and restricted brain field of view have largely hampered the global analysis of the entire brain in detail (Long et al., 2017). In this regard, spatial transcriptomic technologies based on next-generation sequencing, such as spatial transcriptomics (Stahl et al., 2016), Slide-seq (Rodriques et al., 2019), HDST (Vickovic et al., 2019), DBiT-seq (Liu et al., 2020), and Seq-Scope (Cho et al., 2021), have been employed in the field of brain development. However, these methods have limitations in terms of capture sensitivity, resolution, and field of view. To address these issues, we recently developed Stereo-seq, a DNA nanoball (DNB) patterned array-based technology that allows ultra-high resolution and spatial transcriptomics profiling with a large field of view (Chen et al., 2021). Stereo-seq enables the identification of subregions and cellular resolution analysis in complex tissues, and it has been applied to map spatial patterns of gene expression across whole mouse embryos with particular interests in the developing brains from E9.5 to E16.5 (Chen et al., 2021); however, no high-resolution spatial transcriptomic study has been performed on the neonatal developmental stage of the whole mouse brain.

In this study, we applied Stereo-seq to generate a spatially resolved transcriptomic dataset of P7 murine brain sagittal sections near the middle line. Also, we determined the subcellular distribution of 27,330 genes across the entire section. We identified 41 anatomical regions with differentially expressed genes and enriched region-specific gene regulatory networks. Moreover, we performed image-based cell segmentation on this dataset and identified 99,365 cells, with 41 cell types localized in different regions. This study provides a comprehensive resource for future research on the cellular and molecular mechanisms of postnatal brain development. To facilitate this exploration, we created a website containing an open and interactive database (https://db.cngb.org/stomics/datasets/STDS0000139?tab=explore).

## Materials and Methods

### Tissue Collection

In this study, we used P7 C57BL/6 mice purchased from Jiangsu ALF Biotechnology Co., Ltd. (http://jsalfei.com). The methods related to laboratory animals were approved by the Institutional Review Board of the Ethics Committee of BGI (permit no. BGI-IR20210903001). First, the mice were sacrificed via carbon dioxide asphyxiation, and the whole brain was harvested and immediately immersed in embedding molds with precooled Tissue-Tek OCT (Sakura, 4583). Then, the molds with tissue were transferred into prechilled isopentane using liquid nitrogen until the OCT was completely solid. Finally, the embedded tissue was stored at −80°C.

### Tissue Processing and Imaging

Stereo-seq experiments were performed as previously described (Chen et al., 2021). We used a Stereo-seq capture chip with an area of 200 mm^2^ (20 mm × 10 mm). First, the chip was washed with NF-H_2_O supplemented with 0.05 U/µL RNase inhibitor (NEB, M0314L) and dried at room temperature. The embedded tissue was cut to a thickness of 10 µm using a Leika CM1950 cryostat. Cryosections were adhered to the surface of the Stereo-seq capture chip and incubated at 37°C for 5 min. The chip with the section was then fixed in precooled methanol for 40 min at −20°C. Subsequently, the chip was removed and air-dried. The following operation was only used in section 1: 200 µL of tissue fluorescent staining solution (0.1 × SSC (Thermo, AM9770) with 1/200 nucleic acid dye (Thermo Fisher, Q10212) and 2 U/µL RNase inhibitor) was dripped on the chip for 3 min. The staining solution was then removed, and the chip was washed using wash buffer (0.1 × SSC supplemented with 2 U/µL RNase inhibitor), which was also used for the subsequent washing steps. Imaging was performed using a Ti-7 Nikon Eclipse microscope of the FITC channel (objective ×10).

### Stereo-Seq Library Preparation

After washing, 200 µL of a permeabilization reagent (0.1% pepsin (Sigma, P7000) in 0.01 M HCl buffer) was dripped on the chip and incubated at 37°C for 12 min. The reagent was then removed, and the chip was carefully washed. Subsequently, 200 µL RT mix (1 × first-strand buffer, 10 U/µL SuperScript II (Invitrogen, 18064-014), 1 mM dNTPs, 1 M betaine solution, 7.5 mM MgCl_2_, 5 mM DTT, 2 U/µL RNase inhibitor, and 2.5 µM Stereo-seq-TSO (5-CTGCTGACGTACTGAGAGGC/rG//rG//iXNA_G/-3)) were dripped on the chip, which was incubated at 42°C for 2 h. After reverse transcription, the tissue was washed twice with wash buffer and digested with tissue removal buffer (10 mM Tris-HCl, 25 mM EDTA, 100 mM NaCl, and 0.5% SDS) at 37°C for 30 min. The chip was then treated with exonuclease I (NEB, M0293L) for 1 h at 37°C and washed twice with the wash buffer. The resulting first-strand cDNAs were amplified using the KAPA HiFi Hotstart Ready Mix (Roche, KK2602) with a 0.8 µM cDNA–polymerase chain reaction (PCR) primer (5-CTG​CTG​ACG​TAC​TGA​GAG​GC-3), followed by incubation at 95°C for 5 min, 15 cycles at 98°C for 20 s, 58°C for 20 s, and 72°C for 3 min, and a final incubation at 72°C for 5 min.

### Library Construction and Sequencing

The resulting cDNA products were quantified using the Qubit™ dsDNA Assay Kit (Thermo, Q32854) after purification using VAHTS DNA Clean Beads (Vazyme, N411-03, 0.6×). First, 20 ng of products was fragmented using in-house Tn5 transposase at 55°C for 10 min, after which the reaction was stopped by the addition of 0.02% SDS. The fragmentation products were then amplified using KAPA HiFi Hotstart Ready Mix with 0.3 µM Stereo-seq-Library-F primer (/5phos/CTGCTGACGTACTGAGAGG*C*A-3) and 0.3 µM Stereo-seq-Library-R primer (5-GAG​ACG​TTC​TCG​ACT​CAG​CAG​A-3) followed by incubation at 95°C for 5 min, 13 cycles at 98°C for 20 s, 58°C for 20 s, and 72°C for 30 s, and 1 cycle at 72°C for 5 min. Finally, the PCR products were purified using VAHTS DNA Clean Beads (×0.6 and ×0.15). The library was sequenced (35 bp for read 1 and 100 bp for read 2) using an MGI DNBSEQ-Tx sequencer at the China National Gene Bank.

### Processing of Stereo-Seq Raw Data

The fastq files of Stereo-seq, generated by the MGI DNBEQ-Tx sequencer, were processed in accordance with a previous study using the SAW pipeline (https://github.com/BGIResearch/SAW) (Chen et al., 2021). Briefly, the coordinate identity (CID) sequences of DNB were mapped to the designed coordinates of the *in situ* captured chip, which allowed one base mismatch. The unique molecular identifiers (UMIs) containing either N bases or more than two bases with a quality score lower than 10 were filtered out. Next, the CIDs and UMIs were appended to each associated read header of read 2, and read 2 was aligned to the reference genome (mm10) using STAR (Dobin et al., 2013); mapped reads with MAPQ <10 were filtered out. The retained reads were annotated to their corresponding genes. The UMIs with the same CID and gene locus collapsed, allowing one mismatch to be corrected for sequencing and PCR errors. This information was used to generate a CID-containing expression profile matrix.

### Cell Segmentation

The cells were segmented based on nucleic acid staining of the same section. We summed the UMI of each CID, converted the gene matrix into an image where each pixel corresponded to one CID, and manually aligned it with the image of the nucleic acid staining. Then, we kept the image of the nucleic acid staining in the same size as that of the CID, so that each “x” and “y” corresponded to a pixel on the image of the nucleic acid staining. After alignment, we applied the watershed algorithm of the Scikit-image to perform cell segmentation based on nucleic acid staining using a block size of 41 and an offset of 0.003. The cells were then extracted using Euclidean distance transformation with a distance of 15 from the background. Finally, for each segmented cell, the UMI of each gene from different CIDs was aggregated, and a cell matrix was generated for downstream analysis. The differential expressed genes (DEGs) were identified using the FindAllMarker function and were defined as those with a fold change >2, adjusted P value < 0.05, and different positive ratio between case and control >0.1.

### Spatially Constrained Clustering

The expression matrix of the P7 mouse brain was binned into bin 50 (25 µm), and the UMIs of each gene in each bin from different CIDs were aggregated. In a previous study, the expression matrix of bin 50 was used to perform spatially constrained clustering analysis, which included spatial information during unsupervised clustering (Chen et al., 2021). The data were log-normalized in Scanpy (Wolf et al., 2018); highly variable genes were selected, and principal component analysis (PCA) reductions were performed. The spatial k-nearest neighbor (KNN) graph was built using Squidpy (Palla et al., 2022) and then collapsed with the KNN graph of gene expression, which was created using Scanpy with 30 dimensions. The combined graph was used as the input for the Leiden algorithm to identify cell clusters. The marker genes of each cluster were then calculated using Seurat with min.pct = 0.05. Each cluster was annotated based on canonical markers and anatomical annotation of the Allen Brain Atlas (Lein et al., 2007).

### Regulon Analysis

The regulon analysis of transcript factors followed the standard pySCENIC pipeline (Van de Sande et al., 2020). First, the gene expression profiles of bin 50 were used as the input and the GENIE3 algorithm (Huynh-Thu et al., 2010) was used to reconstruct the coexpression network between each transcript factor and the other genes. The coexpression module of each transcription factor with other genes was filtered using the cisTarget database (https://resources.aertslab.org/cistarget/) with default parameters. Next, the regulon activity (area under the curve, AUC) was analyzed using the AUCell function with the default threshold. Finally, the regulon enrichment of each anatomical region was defined as the mean activity of each anatomical region minus the mean activity of all bins and filtering out low regulons with enrichment of less than 0.1 in all anatomical regions.

### Unsupervised Clustering of Segmented Cells

For the analysis of segmented cells in the P7 mouse brain, we filtered out cells with gene numbers less than 300 or those with a total number of detected genes ranking in the top 0.2%. The resulting cells were further processed using Seurat (Hao et al., 2021), followed by SCTransform, scaling, feature gene selection, PCA dimension reduction, and clustering with a resolution parameter of 3. Each cluster was annotated based on the marker genes of cell types.

## Results

### Stereo-Seq Data Quality Control of the P7 Mouse Brain Sections

**FIGURE 1:**
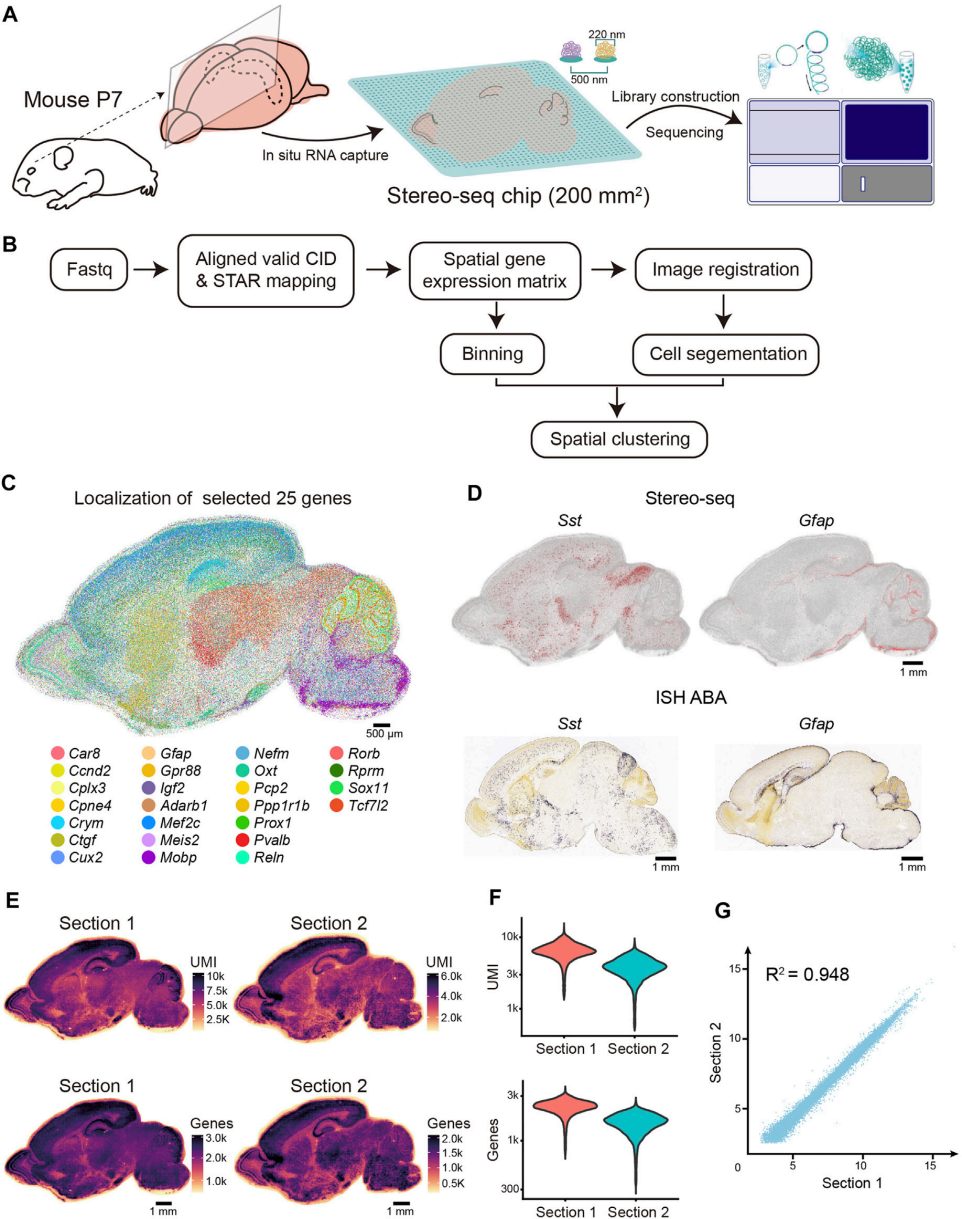
Overview of the experimental method, data analysis workflow, and Stereo-seq data quality. **(A)** Schematic representation of the Stereo-seq workflow. Left, sagittal sections near the middle line were collected for the Stereo-seq profiling. Middle, *in situ* RNA capture from tissue. The effective area of chip in this study is 200 mm^2^. Spot size and center-to-center distance of Stereo-seq chip are 200 nm and 500 nm respectively. Right, library construction and sequencing. **(B)** Graphic representation of Stereo-seq analytical workflow. **(C)** Molecular localization of the 25 selected genes. Each dot represents a captured gene. Scale bar, 500 µm. **(D)** Top: Spatial visualization of *Sst* and *Gfap* localization of the P7 mouse brain section. Bottom: ISH images of *Sst* and *Gfap* localization of the P4 mouse brain section taken from ABA. Scale bars, 1 mm. **(E)** Spatial distribution of UMIs and genes (bin 50) per section. Scale bars, 1 mm. **(F)** Violin plot indicating the distribution of the UMI and genes (bin 50) per section. **(G)** Pearson correlation coefficient (*R* ^2^ = 0.948) between the two replicates.

We generated Stereo-seq libraries of two sagittal sections (sections 1 and 2) near the middle line of the P7 mouse brain (Figure 1A). We obtained 17.944 billion raw fastq reads covering a tissue area of 82.01 and 80.08 mm^2^, respectively (Supplementary Table S1). Simultaneously, we systematically evaluated the quality of the Stereo-seq dataset based on the quality of sequencing, the mapping ratio, the distribution of the known maker genes, the capture efficiency, and the correlation between two sections. First, we evaluated the quality of sequencing data; for section 1, the Q30 in CID was 85.21%, Q30 in UMI was 82.6%, and Q30 in insertion was 83.96% (Supplementary Table S1). The retained reads were processed using the SAW pipeline in accordance with a previous report (Figure 1B). Afterwards, for section 1, 59.3% reads with valid CID were then aligned to genome, with a mapping ratio of 87.1%. Then, the low mapping quality alignments were filtered out according to MAQP. Finally, we obtained 95.35 million CIDs with transcript capturing after registration with the image of the nuclei acid staining (Supplementary Table S1).

Next, to evaluate the spatial gene expression pattern of Stereo-seq, we selected 25 reported regional-specific genes from 27,330 genes to show the spatial expression patterns for section 1 of the P7 mouse brain. The anatomical regions of the brain were observed based on gene expression patterns (Figure 1C). As expected, the distribution of specific brain cell markers (*Sst* and *Gfap*) exhibited remarkable similarity to *in situ* hybridization (ISH) data from P4 mice (Allen Institute) (Lein et al., 2007) (Figure 1D). To evaluate the efficiency of the high-resolution *in situ* capture of Stereo-seq, we calculated the UMIs and gene counts of each bin (bin 50, 50 × 50 DNB bins, 25 µm diameter), which is an appropriate method to identify the different anatomical regions of the Stereo-seq data (Chen et al., 2021; Wu et al., 2021). In section 1, 27,330 genes were detected, with an average of 6,425 UMI and 2,276 genes at bin 50 resolution (Figures 1E,F and Supplementary Table S1). The Pearson correlation coefficient indicated a high correlation between the two sections (*R* ^2^ = 0.948) (Figure 1G). Hence, our Stereo-seq dataset for the P7 mouse brain illustrates the in situ capture of mRNA molecules with high accuracy and efficiency and thus provides a high-quality resource for subsequent data exploration and biological interpretation.

### Region-Specific Patterns of Gene Expression and Gene Regulatory Networks

**FIGURE 2:**
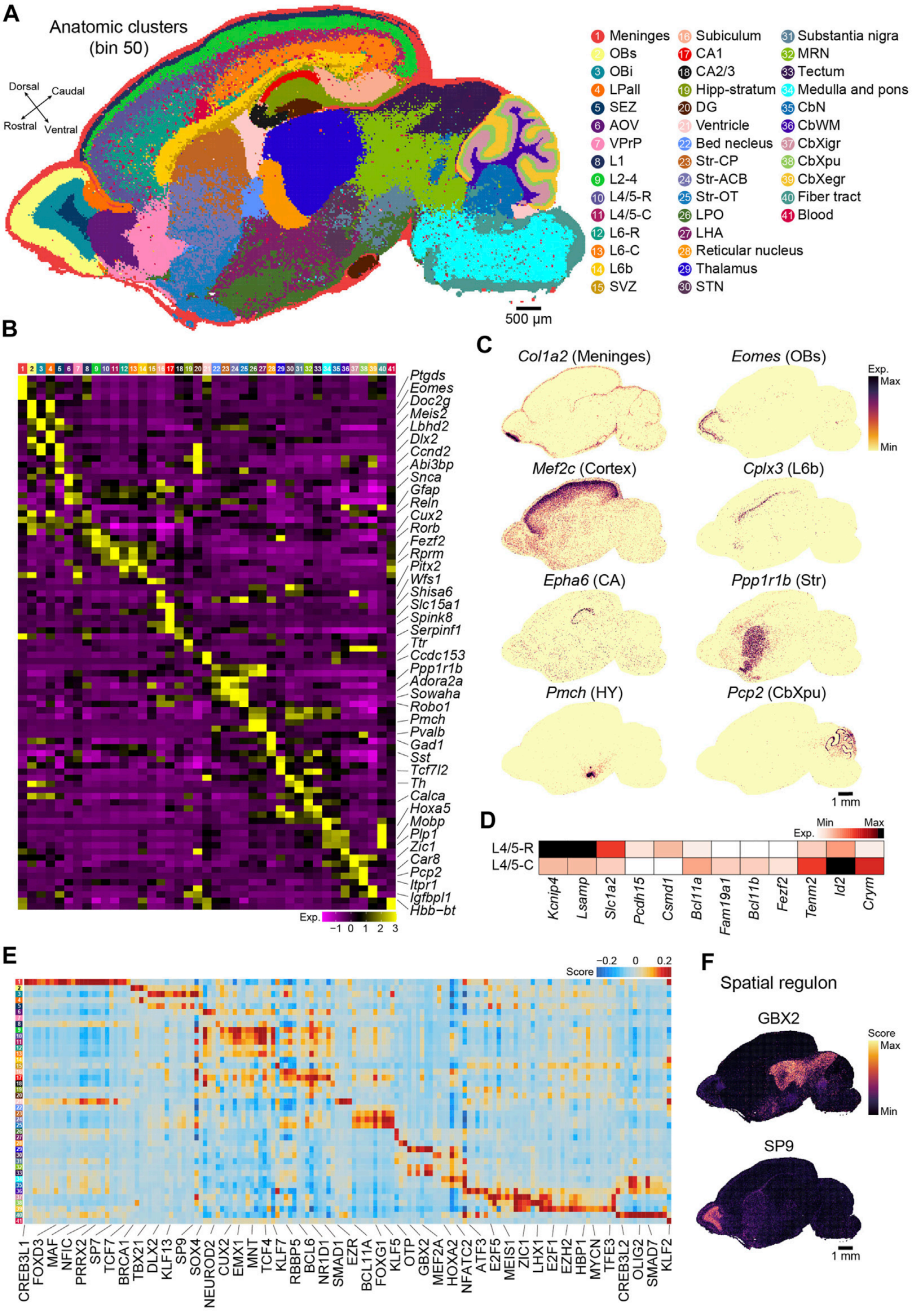
Recognition of regional and molecular characteristics of the anatomical regions based on binning. **(A)** Unsupervised spatially constrained clustering of the P7 mouse brain sagittal section 1 analyzed by Stereo-seq at bin 50 resolution. Bins are colored by their annotation. OBs, superficial stratum of olfactory bulb; OBi, intermediate stratum of olfactory bulb; LPall, lateral pallium; SEZ, subependymal zone; AOV, anterior olfactory nucleus; VPrP, ventropallial prepiriform area; L1, cortical layer 1; L2–4, cortical layers 2–4; L4/5-R, cortical rostral layer 4/5; L4/5-C, cortical caudal layer 4/5; L6-R, cortical rostral layer 6; L6-C, cortical caudal layer 6; L6b, cortex layer 6b; SVZ, subventricular zone; CA1, cornu ammonis area 1; CA2/3, cornu ammonis area 2/3; Hipp-stratum, stratum molecular of CA and DG; DG, dentate gyrus; Str-CP, caudoputamen; Str-ACB, nucleus accumbens; Str-OT, olfactory tubercle; LPO, lateral preoptic area; LHA, lateral hypothalamus; STN, subthalamic nucleus; MRN, midbrain reticular nucleus; CbN, cerebellar nuclei; CbWM, white matter of the cerebellum; CbXigr, internal granular layer of the cerebellum cortex; CbXpu, Purkinje cell layer of cerebellum cortex; CbXegr, external granular layer of cerebellum cortex. Scale bar, 500 µm. **(B)** Heatmap of normalized expression of selected marker genes for the indicated 41 clusters of the P7 mouse brain shown in **(A)**. **(C)** Spatial visualization of the indicated gene expression for meninges (*Col1a2*+), OBs (*Eomes*+), cortex (*Mef2c*+), L6b (*Cplx3*+), CA (*Epha6*+), Str (*Ppp1r1b*+), HY (*Pmch*+), and CbXpu (*Pcp2*+) in the section shown in **(A)**. Scale bar, 1 mm. **(D)** Heatmap of the differential expressed genes between L4/5-R and L4/5-C. **(E)** Heatmap of each cluster’s predicted regulons for the P7 mouse brain section shown in **(A)**. **(F)** Spatial visualization of the representative regulons in **(E)**. Scale bar, 1 mm.

Next, we applied an unsupervised spatially constrained clustering method [(Chen et al., 2021), see Methods] to reconstruct the spatial heterogeneity of the sagittal brain section of P7 mice at bin 50 resolution. We identified 41 anatomical regions that were carefully annotated by canonical markers in each anatomical region (Figures 2A,B and Supplementary Table S2). Tissue-specific identities were confirmed by visualizing the specific marker genes (Figure 2C and Supplementary Figure S1A). The distribution of specific markers (*Tshz1* and *Pvalb*) captured by Stereo-seq exhibited the expected similarity to in situ hybridization (ISH) data taken from BrainTx (Supplementary Figure S1B). The anatomical clusters included 27 clusters in the forebrain, 3 in the midbrain, 7 in the hindbrain, and 2 widely distributed throughout the brain section [meninges (*Ptgds*+)] and blood (*Hbb-bt*+). The forebrain was divided into the main anatomical regions, including the olfactory bulb (OB), neocortex, hippocampus, striatum, and diencephalon. Six subregions across the OB were identified: the superficial stratum of the OB (OBs, *Cdhr1*+), intermediate stratum of the OB (Obi, *Tshz1*+), subependymal zone (SEZ, *Dlx1*+ and *Sox11*+), lateral pallium (LPall, *Fst*+), anterior olfactory nucleus (AOV, *Abi3bp*+ and *Galntl6*+), and ventropallial prepiriform area (VPrP, *Glul*+). For the neocortex, the Stereo-seq dataset was used to identify different cortical layers based on spatially region-specific genes, including layer 1 (L1, *Gfap*+), layers 2–4 (L2-4, *Cux2*+ and *Rorb2*+), layer 4/5 (L4/5, *Rorb*+ and *Fezf2*+), layer 6 (L6, *Rprm*+), layer 6b (L6b, *Cplx3*+ and *Ctgf*+), and the subventricular zone (SVZ, *Dlx1*+, *Dlx2*+, *Sox11*+, and *Ccnd2*+). In addition to the laminar structure, we observed significant differences along the rostral and caudal axes within deep layer, which suggested functional differences along the rostral and caudal axes in the neocortex sublayers. For example, *Lsamp* and *Kcnip4* were highly expressed in L4/5-R, whereas teneurin transmembrane protein 2 (*Tenm2*) was enriched in L4/5-C (Figure 2D). We also identified region-specific gene expression profiles in different subcortical regions, such as the ventricle, which contains the choroid plexus (*Ttr*+), and striatum, including three subregions: caudoputamen (Str-CP, *Ppp1r1b*+ and *Cxcl14*+), nucleus accumbens (Str-ACB, *Ppp1r1b*+ and *Adora2a*+), and olfactory tubercle (Str-OT, *Wfs1*+ and *Robo1*+). In the diencephalon, we observed clusters indicative of the thalamus (*Kitl*+ and *Ntng1*+), reticular nucleus (*Pvalb*+ and *Gad1*+), subthalamic nucleus (STN, *Pitx2*+), lateral hypothalamus (LHA, *Pmch*+), and hypothalamus (HY, *Avp*+ and *Oxt*+). The hippocampus could be subdivided into five subregions: cornu ammonis area 1 (CA1, *Fibcd1*+, *Wfs1*+, and *Shisa6*+), cornu ammonis area 2/3 (CA2/3, *Nptx1*+ and *Spink8*+), dentate gyrus (DG, *Prox1*+ and *Neurod1*+), subiculum (*Nts*+), and stratum molecular of CA and DG (Hipp stratum, *Camk2a*+). In the midbrain, we observed typical subanatomical regions, such as the substantia nigra (*Th*+ and *Gata3*+). The cerebellum was divided into four layers and one nucleus, including the external granular layer (CbXegr, *Cenpa*+), Purkinje cell layer (CbXpu, *Car8*+ and *Pcp2*+), internal granular layer of the cerebellum cortex (CbXigr, *Zic1*+ and *Cbln1*+), white matter of the cerebellum (CbWM, *Gfap+* and *Spp1*+), and cerebellar nuclei (*Plp1+*) (Figures 2A–C; Supplementary Table S2).

We also employed pySCENIC (Van de Sande et al., 2020) to calculate the activity of the gene regulatory network (also known as regulon) and assess the regulatory activity of the transcription factor across all anatomical regions. The regulons showed strong regional specificity (Figure 2E; Supplementary Table S3). For example, GBX2 was enriched in clusters 29 (thalamus) and 32 and 33 (midbrain), which was consistent with previous reports that *Gbx2* is expressed in thalamic postmitotic neuronal progenitors and is essential for the maintenance of thalamic neuronal identity (Figures 2E,F) (Nakayama et al., 2013), as well as that it is a transcriptional repressor that regulates the specification and morphogenesis of the mid-hindbrain junction (Nakayama et al., 2013). SP9, a zinc finger transcription factor, was also highly enriched in cluster 3 (Obi) (Figures 2E,F), which was consistent with its function as a regulator of GABAergic neuron production in the olfactory bulb (Zhang et al., 2016). Our analysis revealed strong heterogeneity in both gene expression and gene regulatory networks across anatomical regions in the postnatal mouse brain and suggested that our Stereo-seq data are a great resource for further exploration of molecular regulation in the brain.

### Spatial Distribution of Cell Types in the P7 Mouse Brain

**FIGURE 3:**
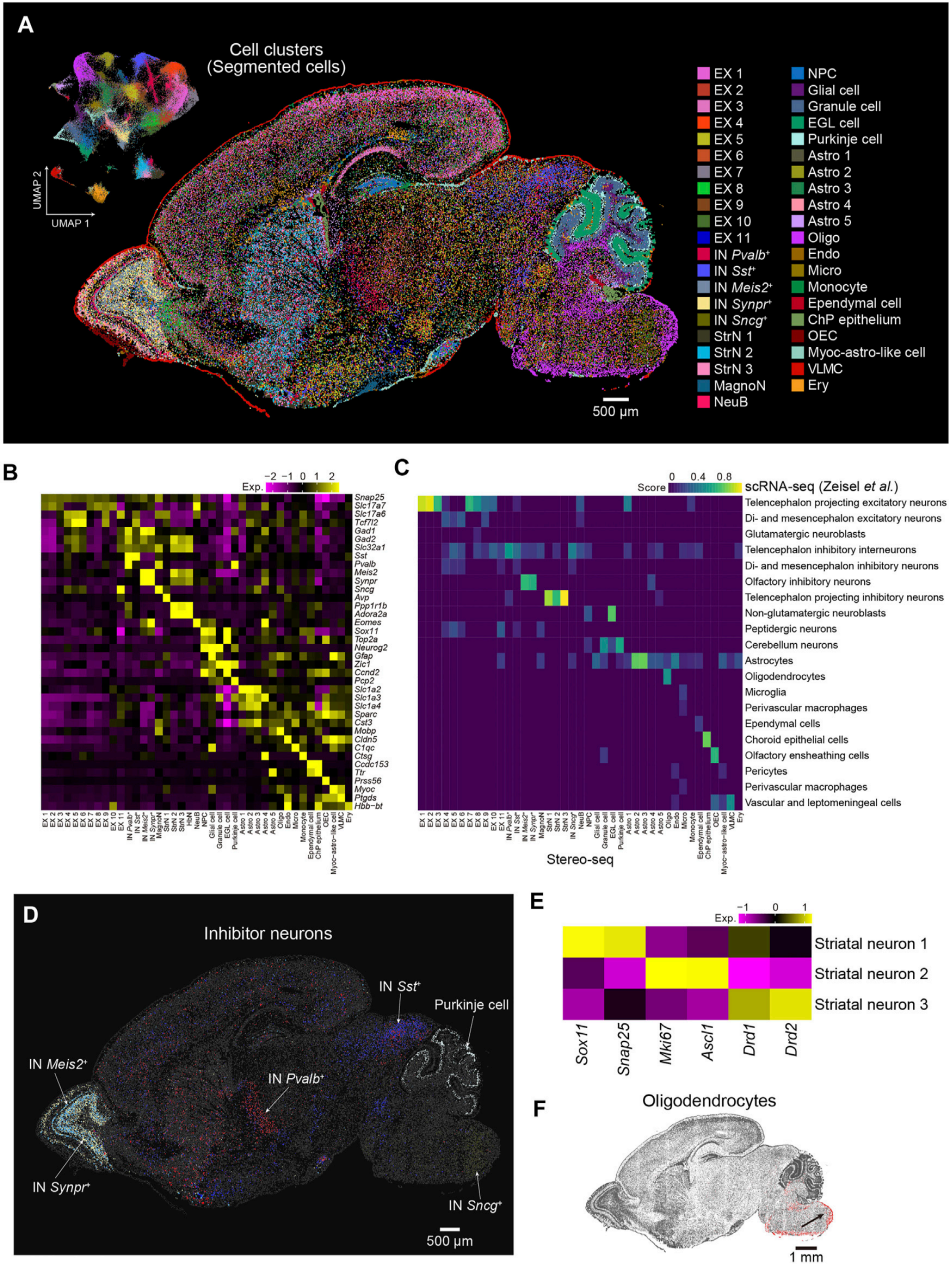
Spatial distribution of cell types in the P7 mouse brain. **(A)** Left: UMAP visualization of the segmented cells from the P7 mouse brain section 1. Right: Spatial visualization of cell types shown in the left panel for the whole brain section 1. Cells are colored by the annotations. EX, excitatory neuron; IN, inhibitory neuron; MagnoN, magnocellular neuron; StrN, striatal neuron; NeuB, neuroblast; NPC, neuron progenitor cell; EGL cell, external granular layer cell; Astro, astrocyte; Oligo, oligodendrocyte; Endo, endothelium; Micro, microglia; ChP epithelium, choroid plexus epithelium; OEC, olfactory ensheathing cell; Myoc-astro-like cell, myoc-expressing astrocyte-like cell; VLMC, vascular leptomeningeal cell; Ery, erythrocyte. Scale bar, 500 µm. **(B)** Heatmap of normalized expression of selected marker genes for the 41 cell types of the P7 mouse brain shown in **(A)**. **(C)** Correspondence between the cell types shown in A by Stereo-seq and the cell clusters identified by reported scRNA-seq (Zeisel et al., 2018). **(D)** Spatial visualization of different subtypes of inhibitor neuron clusters shown in **(A)**. Scale bar, 500 µm. **(E)** Heatmap of normalized expression of the indicated genes for the three striatal neuron types shown in **(A)**. **(F)** Spatial visualization of oligodendrocytes shown in **(A)**. Scale bar, 1 mm.

Cells are fundamental elements of organs. The high resolution of the Stereo-seq technology enables the dissection of the spatial transcriptome at cellular resolution at the whole section scale. Next, we performed cell segmentation analysis by registration of the image of the nucleic acid staining and Stereo-seq data using Scikit-image. Segmented cells with gene number less than 300 or the top 0.2% cells ranked by gene number were filtered out. A total of 99,365 cells with an average number of 787 UMI and 698 genes per cell were used for downstream analysis. Subsequently, we employed Seurat to evaluate the cell heterogeneity of sagittal brain section 1 at cellular resolution and identified 41 major cell types that could be annotated using known putative markers, including 25 clusters of neurons and 16 clusters of nonneuronal cells (Figures 3A,B). These annotations were further confirmed by correlation analysis using a publicly available scRNA-seq dataset (Zeisel et al., 2018) (Figure 3C). By the analysis of differential expressed genes (DEGs), we obtained 505 DEGs across the 41 clusters (Supplementary Figure S2). For example, *Sostdc1* and *Calml4* were highly expressed in the choroid plexus epithelium (ChP epithelium). Olfactory ensheathing cell (OEC) highly expressed *Rcn3* and *Lum*. *Gm20425* and *Grb14* were highly enriched in oligodendrocyte (OLI). The spatial distribution of the cell types showed strong regional heterogeneity, which indicates specific functions in each region. For example, *Meis2*+ and *Synpr*+ inhibitory neurons were located in the olfactory bulb, and the distribution of the *Meis2*+ inhibitory neurons was wider. *Sst*+ inhibitory neurons were highly enriched in the inferior colliculus of the midbrain, whereas *Pvalb*+ inhibitory neurons were mostly enriched in the reticular nucleus and striatum. *Sncg*+ inhibitory neurons were mainly located in the medullary region of the hindbrain, and Purkinje cells were located in the cerebellum (Figure 3D). We identified three neuronal subclusters in the striatum, which widely expressed *Ppp1r1b* and *Adora2a*. The striatal neuron 1 cluster displayed high expression of *Sox11*, an immature neuron marker (Bergsland et al., 2006; Chen et al., 2015), whereas striatal neuron 2 expressed both proliferation (*Mki67*) and progenitor (*Ascl1*) markers. Striatal neuron 3 was characterized by high expression levels of genes related to GABAergic spiny projection neurons that receive and integrate information within the striatum, such as *Drd1* and *Drd2* (Anderson et al., 2020) (Figure 3E). We also observed that oligodendrocytes mainly appeared in the hindbrain, which suggested that neurons in the hindbrain were myelinated at this stage (Figure 3F). Overall, our results demonstrate the utility of Stereo-seq data to comprehensively characterize cell types and cell state heterogeneity at the whole-brain section level, which is paramount for understanding cell differentiation processes involving regional and subregional specificities.

## Conclusion

We report a high-resolution spatial transcriptomic atlas of sagittal sections from the P7 mouse brain using Stereo-seq. This dataset enables the exploration of the spatial gene expression patterns of 27,330 genes across the medial structures near the midline of the postnatal mouse brain. By applying unsupervised clustering, we identified 41 anatomical regions with differentially expressed genes and showed enrichment of gene regulatory networks, which may be associated with the functions of different regions. We also provided a single-cell resolution map of the whole brain section using image-based cell segmentation and identified 99,365 cells of 41 subtypes in different regions of the P7 mouse brain section. This dataset provides a comprehensive spatial map of anatomical regions, genes, regulons, and cell types in the P7 mouse brain and has significant implications for studies on cell differentiation, cell migration, intercellular interactions, and the underlying regulatory mechanisms. In addition, our work paves the way for studies on brain developmental disorders.

## Data Availability

The datasets presented in this study can be found in online repositories. The names of the repository/repositories and accession number(s) can be found in the article/Supplementary Material.

## Supplementary Material

Supplementary Figure S1 Spatial visualization of the indicated gene expression in the section shown in Figure 2A.

Supplementary Figure S2 Heatmap of the differential expressed genes of the clusters in Figure 3A.

## References

[B1] Anderson A. G. Kulkarni A. Harper M. Konopka G. (2020). Single-Cell Analysis of Foxp1-Driven Mechanisms Essential for Striatal Development. Cel Rep. 30, 3051–3066. 10.1016/j.celrep.2020.02.030 PMC7137930 32130906

[B2] Bakken T. E. Miller J. A. Ding S.-L. Sunkin S. M. Smith K. A. Ng L. (2016). A Comprehensive Transcriptional Map of Primate Brain Development. Nature 535, 367–375. 10.1038/nature18637 27409810 PMC5325728

[B3] Bergsland M. Werme M. Malewicz M. Perlmann T. Muhr J. (2006). The Establishment of Neuronal Properties Is Controlled by Sox4 and Sox11. Genes Dev. 20, 3475–3486. 10.1101/gad.403406 17182872 PMC1698453

[B4] Carter R. A. Bihannic L. Rosencrance C. Hadley J. L. Tong Y. Phoenix T. N. (2018). A Single-Cell Transcriptional Atlas of the Developing Murine Cerebellum. Curr. Biol. 28, 2910–2920. 10.1016/j.cub.2018.07.062 30220501

[B5] Chen C. Lee G. A. Pourmorady A. Sock E. Donoghue M. J. (2015). Orchestration of Neuronal Differentiation and Progenitor Pool Expansion in the Developing Cortex by SoxC Genes. J. Neurosci. 35, 10629–10642. 10.1523/jneurosci.1663-15.2015 26203155 PMC4510297

[B6] Chen A. Liao S. Cheng M. Ma K. Wu L. Lai Y. (2021). Spatiotemporal Transcriptomic Atlas of Mouse Organogenesis Using DNA Nanoball Patterned Arrays. bioRxiv, 427004. 10.1101/2021.01.17.427004 35512705

[B7] Cho C.-S. Xi J. Si Y. Park S.-R. Hsu J.-E. Kim M. (2021). Microscopic Examination of Spatial Transcriptome Using Seq-Scope. Cell 184, 3559–3572. 10.1016/j.cell.2021.05.010 34115981 PMC8238917

[B8] Di Bella D. J. Habibi E. Stickels R. R. Scalia G. Brown J. Yadollahpour P. (2021). Molecular Logic of Cellular Diversification in the Mouse Cerebral Cortex. Nature 595, 554–559. 10.1038/s41586-021-03670-5 34163074 PMC9006333

[B9] Ding S. L. Royall J. J. Lesnar P. Facer B. A. C. Smith K. A. Wei Y. (2022). Cellular Resolution Anatomical and Molecular Atlases for Prenatal Human Brains. J. Comp. Neurol. 530, 6–503. 10.1002/cne.25243 34525221 PMC8716522

[B10] Dobin A. Davis C. A. Schlesinger F. Drenkow J. Zaleski C. Jha S. (2013). STAR: Ultrafast Universal RNA-Seq Aligner. Bioinformatics 29, 15–21. 10.1093/bioinformatics/bts635 23104886 PMC3530905

[B11] Echevarria D. Vieira C. Gimeno L. Martínez S. (2003). Neuroepithelial Secondary Organizers and Cell Fate Specification in the Developing Brain. Brain Res. Brain Res. Rev. 43, 179–191. 10.1016/j.brainresrev.2003.08.002 14572913

[B12] Han X. Wu X. Chung W.-Y. Li T. Nekrutenko A. Altman N. S. (2009). Transcriptome of Embryonic and Neonatal Mouse Cortex by High-Throughput RNA Sequencing. Proc. Natl. Acad. Sci. U.S.A. 106, 12741–12746. 10.1073/pnas.0902417106 19617558 PMC2722358

[B13] Hao Y. Hao S. Andersen-Nissen E. Mauck W. M. 3rd Zheng S. Butler A. (2021). Integrated Analysis of Multimodal Single-Cell Data. Cell 184, 3573–3587. 10.1016/j.cell.2021.04.048 34062119 PMC8238499

[B14] Hodge R. D. Hevner R. F. (2011). Expression and Actions of Transcription Factors in Adult Hippocampal Neurogenesis. Devel. Neurobio. 71, 680–689. 10.1002/dneu.20882 PMC3134120 21412988

[B15] Huynh-Thu V. A. Irrthum A. Wehenkel L. Geurts P. (2010). Inferring Regulatory Networks from Expression Data Using Tree-Based Methods. PLoS One 5, e12776. 10.1371/journal.pone.0012776 20927193 PMC2946910

[B16] Jiang X. Nardelli J. (2016). Cellular and Molecular Introduction to Brain Development. Neurobiol. Dis. 92, 3–17. 10.1016/j.nbd.2015.07.007 26184894 PMC4720585

[B17] Kozareva V. Martin C. Osorno T. Rudolph S. Guo C. Vanderburg C. (2021). A Transcriptomic Atlas of Mouse Cerebellar Cortex Comprehensively Defines Cell Types. Nature 598, 214–219. 10.1038/s41586-021-03220-z 34616064 PMC8494635

[B18] La Manno G. Siletti K. Furlan A. Gyllborg D. Vinsland E. Mossi Albiach A. (2021). Molecular Architecture of the Developing Mouse Brain. Nature 596, 92–96. 10.1038/s41586-021-03775-x 34321664

[B19] Lake B. B. Ai R. Kaeser G. E. Salathia N. S. Yung Y. C. Liu R. (2016). Neuronal Subtypes and Diversity Revealed by Single-Nucleus RNA Sequencing of the Human Brain. Science 352, 1586–1590. 10.1126/science.aaf1204 27339989 PMC5038589

[B20] Lein E. S. Hawrylycz M. J. Ao N. Ayres M. Bensinger A. Bernard A. (2007). Genome-wide Atlas of Gene Expression in the Adult Mouse Brain. Nature 445, 168–176. 10.1038/nature05453 17151600

[B21] Lein E. Borm L. E. Linnarsson S. (2017). The Promise of Spatial Transcriptomics for Neuroscience in the Era of Molecular Cell Typing. Science 358, 64–69. 10.1126/science.aan6827 28983044

[B22] Li M. Santpere G. Imamura Kawasawa Y. Evgrafov O. V. Gulden F. O. Pochareddy S. (2018). Integrative Functional Genomic Analysis of Human Brain Development and Neuropsychiatric Risks. Science 362, eaat7615. 10.1126/science.aat7615 30545854 PMC6413317

[B23] Liao J. Lu X. Shao X. Zhu L. Fan X. (2021). Uncovering an Organ's Molecular Architecture at Single-Cell Resolution by Spatially Resolved Transcriptomics. Trends Biotechnol. 39, 43–58. 10.1016/j.tibtech.2020.05.006 32505359

[B24] Liu Y. Yang M. Deng Y. Su G. Enninful A. Guo C. C. (2020). High-Spatial-Resolution Multi-Omics Sequencing via Deterministic Barcoding in Tissue. Cell 183, 1665–1681. 10.1016/j.cell.2020.10.026 33188776 PMC7736559

[B25] Long X. Colonell J. Wong A. M. Singer R. H. Lionnet T. (2017). Quantitative mRNA Imaging throughout the Entire Drosophila Brain. Nat. Methods 14, 703–706. 10.1038/nmeth.4309 28581495

[B26] Manuel M. N. Mi D. Mason J. O. Price D. J. (2015). Regulation of Cerebral Cortical Neurogenesis by the Pax6 Transcription Factor. Front. Cel. Neurosci. 9, 70. 10.3389/fncel.2015.00070 PMC4354436 25805971

[B27] Martínez S. (2001). The Isthmic Organizer and Brain Regionalization. Int. J. Dev. Biol. 45, 367–371. 10.1387/IJDB.11291867 11291867

[B28] Miller J. A. Ding S.-L. Sunkin S. M. Smith K. A. Ng L. Szafer A. (2014). Transcriptional Landscape of the Prenatal Human Brain. Nature 508, 199–206. 10.1038/nature13185 24695229 PMC4105188

[B29] Nakayama Y. Kikuta H. Kanai M. Yoshikawa K. Kawamura A. Kobayashi K. (2013). Gbx2 Functions as a Transcriptional Repressor to Regulate the Specification and Morphogenesis of the Mid-hindbrain junction in a Dosage- and Stage-dependent Manner. Mech. Develop. 130, 532–552. 10.1016/j.mod.2013.07.004 23933069

[B30] Ortiz C. Carlén M. Meletis K. (2021). Spatial Transcriptomics: Molecular Maps of the Mammalian Brain. Annu. Rev. Neurosci. 44, 547–562. 10.1146/annurev-neuro-100520-082639 33914592

[B31] Palla G. Spitzer H. Klein M. Fischer D. Schaar A. C. Kuemmerle L. B. (2022). Squidpy: a Scalable Framework for Spatial Omics Analysis. Nat. Methods 19, 171. 10.1038/s41592-021-01358-2 35102346 PMC8828470

[B32] Parenti I. Rabaneda L. G. Schoen H. Novarino G. (2020). Neurodevelopmental Disorders: From Genetics to Functional Pathways. Trends Neurosci. 43, 608–621. 10.1016/j.tins.2020.05.004 32507511

[B33] Rodriques S. G. Stickels R. R. Goeva A. Martin C. A. Murray E. Vanderburg C. R. (2019). Slide-seq: A Scalable Technology for Measuring Genome-wide Expression at High Spatial Resolution. Science 363, 1463–1467. 10.1126/science.aaw1219 30923225 PMC6927209

[B34] Stahl P. L. Salmén F. Vickovic S. Lundmark A. Navarro J. F. Magnusson J. (2016). Visualization and Analysis of Gene Expression in Tissue Sections by Spatial Transcriptomics. Science 353, 78–82. 10.1126/science.aaf2403 27365449

[B35] Stiles J. Jernigan T. L. (2010). The Basics of Brain Development. Neuropsychol. Rev. 20, 327–348. 10.1007/s11065-010-9148-4 21042938 PMC2989000

[B36] Tan L. Ma W. Wu H. Zheng Y. Xing D. Chen R. (2021). Changes in Genome Architecture and Transcriptional Dynamics Progress Independently of Sensory Experience during post-natal Brain Development. Cell 184, 741–758. 10.1016/j.cell.2020.12.032 33484631

[B37] Tasic B. Menon V. Nguyen T. N. Kim T. K. Jarsky T. Yao Z. (2016). Adult Mouse Cortical Cell Taxonomy Revealed by Single Cell Transcriptomics. Nat. Neurosci. 19, 335–346. 10.1038/nn.4216 26727548 PMC4985242

[B38] Tasic B. Yao Z. Graybuck L. T. Smith K. A. Nguyen T. N. Bertagnolli D. (2018). Shared and Distinct Transcriptomic Cell Types across Neocortical Areas. Nature 563, 72–78. 10.1038/s41586-018-0654-5 30382198 PMC6456269

[B39] Thompson C. L. Ng L. Menon V. Martinez S. Lee C.-K. Glattfelder K. (2014). A High-Resolution Spatiotemporal Atlas of Gene Expression of the Developing Mouse Brain. Neuron 83, 309–323. 10.1016/j.neuron.2014.05.033 24952961 PMC4319559

[B40] Tiklova K. Björklund Å. K. Lahti L. Fiorenzano A. Nolbrant S. Gillberg L. (2019). Single-cell RNA Sequencing Reveals Midbrain Dopamine Neuron Diversity Emerging during Mouse Brain Development. Nat. Commun. 10, 581. 10.1038/s41467-019-08453-1 30718509 PMC6362095

[B41] Van de Sande B. Flerin C. Davie K. De Waegeneer M. Hulselmans G. Aibar S. (2020). A Scalable SCENIC Workflow for Single-Cell Gene Regulatory Network Analysis. Nat. Protoc. 15, 2247–2276. 10.1038/s41596-020-0336-2 32561888

[B42] Vickovic S. Eraslan G. Salmén F. Klughammer J. Stenbeck L. Schapiro D. (2019). High-definition Spatial Transcriptomics for *In Situ* Tissue Profiling. Nat. Methods 16, 987–990. 10.1038/s41592-019-0548-y 31501547 PMC6765407

[B47] Wolf F. A. Angerer P. Theis F. J. (2018). SCANPY: Large-Scale Single-Cell Gene Expression Data Analysis. Genome Biol. 19, 15. 29409532 10.1186/s13059-017-1382-0 PMC5802054

[B43] Wu L. Yan J. Bai Y. Chen F. Xu J. Zou X. (2021). Spatially-resolved Transcriptomics Analyses of Invasive Fronts in Solid Tumors. bioRxiv, 465135. 10.1101/2021.10.21.465135

[B44] Zeisel A. Hochgerner H. Lönnerberg P. Johnsson A. Memic F. van der Zwan J. (2018). Molecular Architecture of the Mouse Nervous System. Cell 174, 999–1014. 10.1016/j.cell.2018.06.021 30096314 PMC6086934

[B45] Zhang J. Miller M. I. Plachez C. Richards L. J. Yarowsky P. van Zijl P. (2005). Mapping Postnatal Mouse Brain Development with Diffusion Tensor Microimaging. Neuroimage 26, 1042–1051. 10.1016/j.neuroimage.2005.03.009 15961044

[B46] Zhang Q. Zhang Y. Wang C. Xu Z. Liang Q. An L. (2016). The Zinc Finger Transcription Factor Sp9 Is Required for the Development of Striatopallidal Projection Neurons. Cel Rep. 16, 1431–1444. 10.1016/j.celrep.2016.06.090 PMC4972643 27452460

