# Supplementary figures and images for: A Cellular Resolution Spatial Transcriptomic Landscape of the Medial Structures in Postnatal Mouse Brain
Source: Front Cell Dev Biol. 2022 May 17;10:878346. doi: 10.3389/fcell.2022.878346 (PMC9152126; doi:10.3389/fcell.2022.878346)

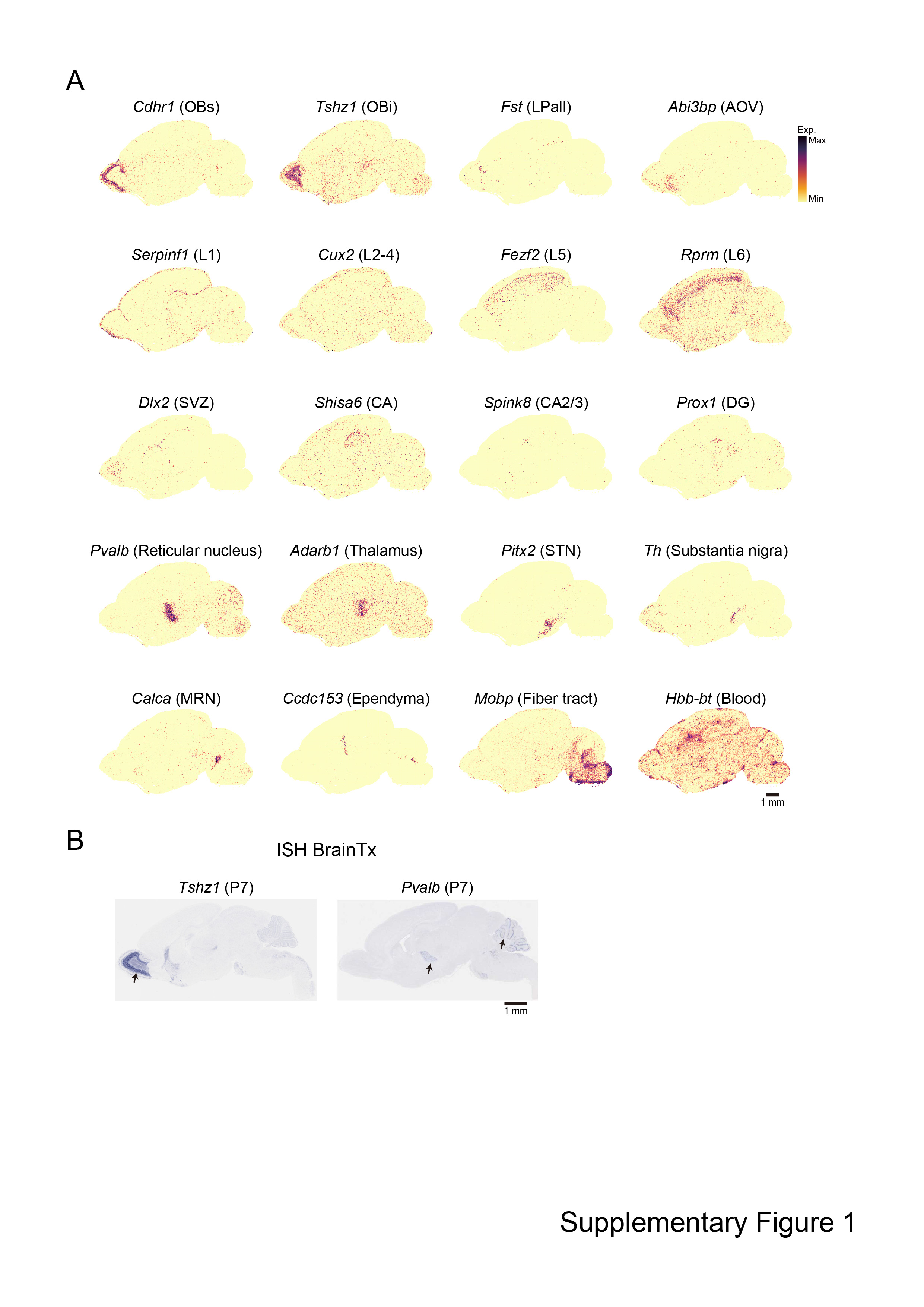

Supplement: Supplementary file 3 [file Image1.jpeg]

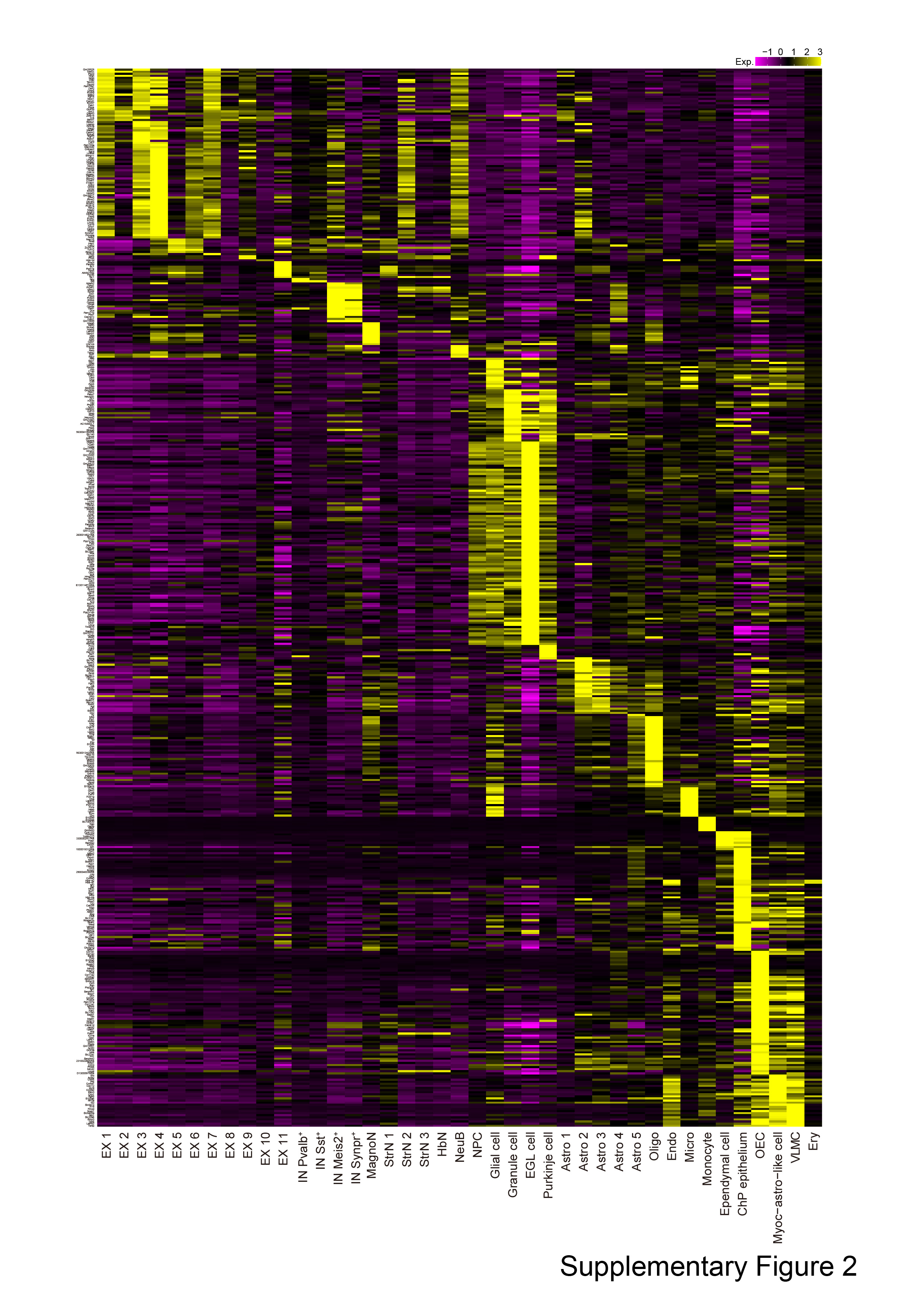

Supplement: Supplementary file 4 [file Image2.jpeg]
